# Adenoid Cystic Carcinoma of the Breast in a Male Patient: A Case Report and Literature Review

**DOI:** 10.3389/fonc.2022.905997

**Published:** 2022-07-07

**Authors:** Dan Wan, Hongyuan Zhou, Yutao Zhang

**Affiliations:** Department of Pathology, The First People’s Hospital of Zigong, Zigong, China

**Keywords:** male breast cancer, adenoid cystic carcinoma, MYB, immunohistochemistry, differential diagnosis

## Abstract

Adenoid cystic carcinoma (ACC) of the breast (breast ACC) is a rare tumor, especially in men, with only 17 cases reported in the literature. Owing to this rarity, male breast ACC is susceptible to missed or incorrect diagnoses, and data on treatment options and prognosis is also scarce. Herein, we report a case of a male patient with primary breast ACC and performed a detailed clinicopathological analysis of the 17 cases reported in the literature. A 38-year-old Chinese man patient developed right-sided breast nipple retraction in 2013 and presented to our hospital in 2015 with a palpable mass in the right breast for four days. B-scan ultrasound indicated the presence of a solid space-occupying lesion in the right breast. Breast Imaging Reporting and Data System (BI-RADS) classified the lesion as category 4B, and mammography showed a right breast nodule classified as BI-RADS 4C. Modified radical mastectomy for breast cancer was performed on the right breast. Microscopic examination of the excised tissue revealed diffuse tumor invasion of the subcutaneous fibers and adipose tissue, with tumor cells arranged in cribriform, tubular, and microcystic patterns. Immunohistochemical staining indicated that the glandular epithelial cells were positive for CD117, CK7, and Ki67 (approximately 30%) and negative for estrogen receptor, progesterone receptor, and human epidermal growth factor receptor 2, while the myoepithelial/basal cells were positive for P63, CK5/6 and S-100. Moreover, basement membrane materials were positive for collagen type IV. Molecular pathology analysis by fluorescence *in situ* hybridization revealed that the tumor was negative for *MYB* rearrangements. The patient was followed up for 82 months with no tumor recurrence or metastasis. According to the current literature, mastectomies have a better prognosis than lumpectomy. Accurately identifying the diagnosis of male breast ACC and considering the surgery of mastectomy may be the key factors for patients to obtain a good prognosis based on the microscopic characteristics of the tumor.

## Introduction

Adenoid cystic carcinoma (ACC) of the breast (breast ACC) is an uncommon subtype of invasive carcinoma, accounting for less than 0.1% of the primary breast carcinomas (1). The disease predominantly affects older women (2) and typically occurs in the upper outer quadrant (3), below the areola, or the central region of the breast (4). The tumor size ranges from 0.1 to 16 cm, with a mean diameter of 2 cm (3). Breast ACC has three histological subtypes: cribriform, tubular, and solid. The cribriform subtype is the most common, characterized by the formation of cancer nests containing true and pseudo cystic spaces by glandular epithelial and myoepithelial/basal cells (2). Based on extensive molecular and genetic profiling studies, a glandular epithelial and a myoepithelial/basal cell population with divergent immunophenotypical patterns (4). The glandular epithelial cells are positive for CK7, CK8/18, and CD117 (c-Kit). On the other hand, the myoepithelial/basal cells are immunoreactive for myoepithelial markers (p63, actin, calponin, S-100 protein) and basal cytokeratins (CK5, CK5/6, CK14, CK17) (1). Most cases of breast ACC exhibit the triple-negative immunophenotype—that is, negative for estrogen receptor (ER), progesterone receptor (PR), and human epidermal growth factor receptor 2 (HER2) (4). Basement membrane materials that were positive for collagen type IV were confirmed around tumor cells (1). The t (6;9) (q22–23; p23–24) chromosomal translocation frequently occurs in breast ACC and salivary gland ACC, resulting in the fusion of the proto-oncogene *MYB* and the transcription factor gene *NFIB*. Previous studies have shown that *MYB* rearrangements can be detected in approximately 22.6% of the patient with breast ACC (5, 6). *MYB*-*NFIB* gene fusion or *MYB* rearrangement supports the diagnosis of ACC. Because of the low incidence of lymph node metastasis in breast ACC, local recurrences and distant metastases are relatively rare, and the disease usually carries a favorable prognosis (2).

Typically, in other countries, the patients with triple-negative breast ACC are treated using conservative methods, such as breast-conserving surgery, while modified radical resection is often used in China (7, 8). Breast ACC is extremely rare in male patients, with only 17 cases reported worldwide (9–25), making the disease highly susceptible to missed or incorrect diagnoses. In addition, evidence-based recommendations are currently lacking regarding medications and surgical approaches. Therefore, the diagnosis and treatment of male breast ACC are often reliant on guidelines for female patients.

Herein, we report a case of a patient with breast ACC in a middle-aged man, followed by a review of the previous literature, to analyze the clinicopathological characteristics of the disease and determine the essentials of its diagnosis and differential diagnosis. By presenting our experience and a review of the current literature, we hope to provide scientific evidence for the diagnosis and treatment of male patients with breast ACC.

## Case Description

A 38-year-old man developed a nipple retraction in the right breast with mild pain and no skin redness. The nipple retraction developed in 2013, but he did not seek medical attention. The condition gradually aggravated until 2015, when he presented to our hospital. The patient had no significant medical history.

Physical examination upon admission revealed nipple retraction in the right breast and a palpable firm mass beneath the nipple, measuring approximately 1.0 × 2.0 cm, with irregular morphology, indistinct boundaries, and poor mobility. B-scan ultrasound indicated the presence of a solid space-occupying lesion in the right breast, which was classified as Breast Imaging Reporting and Data System (BI-RADS) category 4B. Mammography showed right nipple retraction and a dense nodule measuring approximately 1.7 × 1.4 cm beneath the right nipple, with indistinct boundaries, which was classified as BI-RADS 4C. The head and neck computed tomography scans were unremarkable.

## Diagnostic Assessment

**Figure 1:**
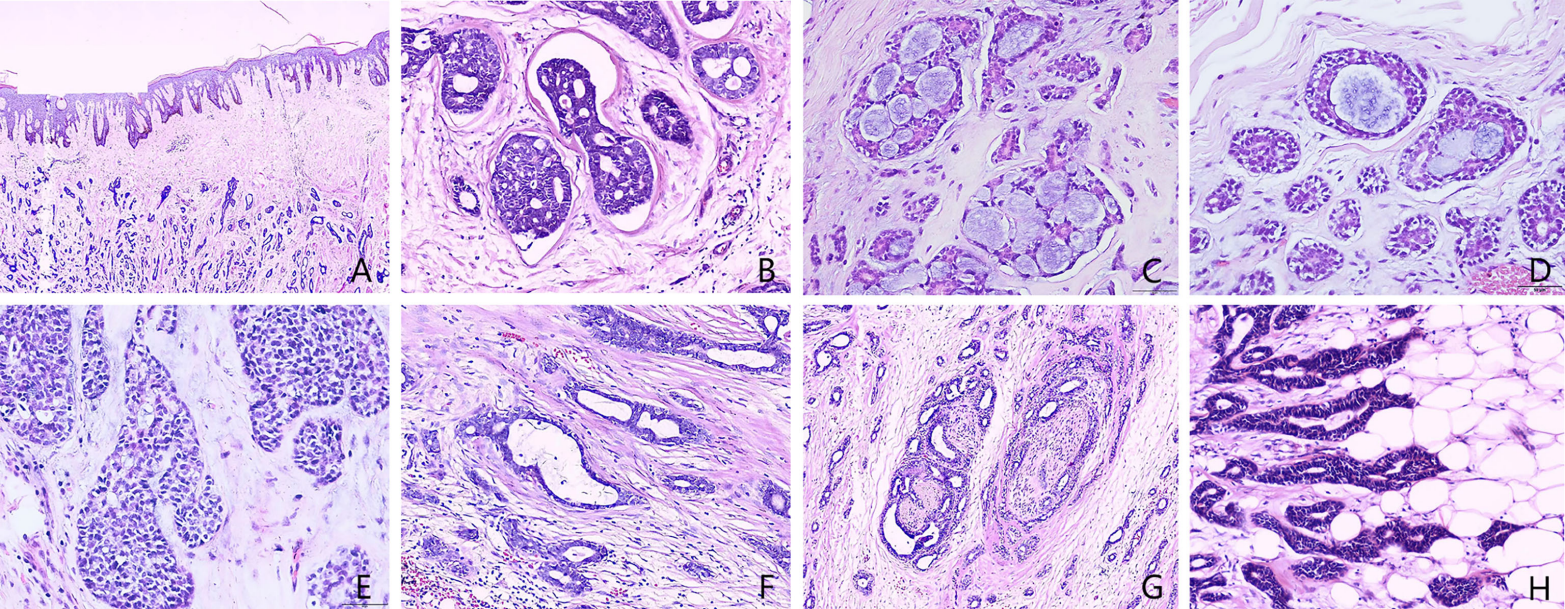
Histopathology of male breast adenoid cystic carcinoma (Hematoxylin and eosin [H&E] stain). **(A)** Microscopic examination of the excised tissue revealed diffuse tumor invasion of the subcutaneous fibers and adipose tissue. **(B)** Cribriform patterns and basement membrane materials around tumor nest. **(C)** Cribriform patterns and the cavities contain mucus secretion. **(D)** Tubular pattern and the cavities contain mucus secretion. **(E)** Solid patterns. **(F)** Microcystic patterns. **(G)** Neural invasion. **(H)** Adipose invasion. [**A**: 40× magnification; **B–H**: 400× magnification].

**Figure 2:**
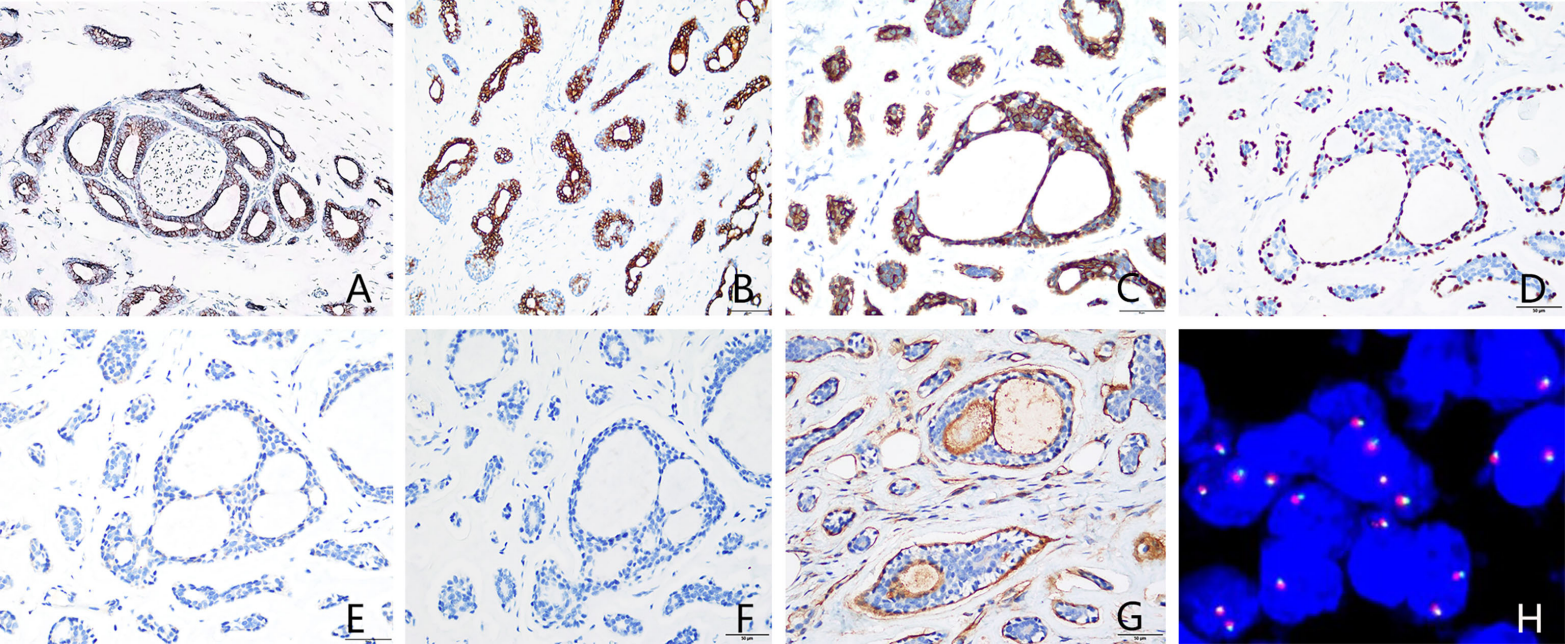
Immunohistochemical staining and *MYB* gene detection. **(A)** Epithelial cells were CD117-positive. **(B)** Epithelial cells were CK7-positive. **(C)** Basal cells were CK5/6-positive. **(D)** P63 highlighted intact myoepithelial cells around the acini. **(E)** ER was negative. **(F)** PR was negative. **(G)** Basement membrane materials were highlighted by collagen type IV staining. **(H)** Molecular pathology analysis by fluorescence *in situ* hybridization (FISH) revealed that the tumor was negative for *MYB* rearrangements. [**(A–G)** immunohistochemical staining, 400× magnification; **(H)** FISH, 1000× magnification]. ER, estrogen receptor; PR, progesterone receptor.

On March 26, 2015, modified radical mastectomy for breast cancer and axillary lymph node dissection were performed on the right breast. Mastectomy specimens were evaluated postoperatively. The gross specimen revealed the presence of a solid, firm tumor measuring approximately 1.5 × 1.0 × 1.0 cm, characterized by indistinct boundaries with the surrounding tissue and a grayish-white cut surface. Freezing slice pathologic diagnosis: Invasive breast cancer. Microscopic examination of the excised tissue revealed diffuse tumor invasion of the subcutaneous fibers and adipose tissue (**Figure 1A**), with tumor cells arranged in cribriform (**Figures 1B, C**), tubular (**Figure 1D**), solid (**Figure 1E**), and microcystic (**Figure 1F**) patterns. Double-layered structures comprising glandular epithelial and myoepithelial/basal cells were found within each type of tumor cell pattern, with the glandular epithelial cells exhibiting a cuboidal shape, acidophilic cytoplasm, round nucleus, and occasional nucleoli. The myoepithelial/basal cells of the outer layer exhibited a spindle shape, low cytoplasmic volume, intense nuclear staining, and sporadic mitosis. There were basement membrane materials around tumor cells, and the tumor cavity contained mucus. Neural (**Figure 1G**) and adipose tissue (**Figure 1H**) invasion was also observed. The results of immunohistochemical staining (**Figures 2A–G**) were as follows: (i) glandular epithelial cells: E-cadherin(+), P53(+), CK7(+), CD117(+), ER(−), PR(−), HER2(−), Ki67(+, approximately 30%); (ii) myoepithelial/basal cells: P63(+), CK5/6(+), S-100(+), smooth muscle actin-negative [SMA(−)], calponin(−), glial fibrillary acidic protein-negative [GFAP(−)]; (iii) basement membrane materials: collagen type IV(+). Molecular pathology analysis by fluorescence *in situ* hybridization (FISH) revealed that the tumor was negative for *MYB* rearrangements (**Figure 2H**). Based on these morphological observations and immunohistochemical staining results, a diagnosis of ACC was preliminarily considered. After consultation with a team of experts from the Military General Hospital of Beijing People’s Liberation Army, the diagnosis of right breast ACC was confirmed. The surgical wound was mildly infected, with redness and exudation. Anti-infective treatment was administered, and the patient was discharged three weeks after the operation. No radiotherapy or chemotherapy was administered postoperatively. He remained healthy over the 82 months of regular follow-up without developing tumor recurrence or metastasis.

## Discussion

Breast ACC predominantly affects women aged 39–73 years (median age: 60.5 years), especially postmenopausal women (2). Primary ACC of the male breast is extremely rare. Based on the present and previously reported cases (**Table 1**), we analyzed the clinicopathological characteristics, diagnosis, and differential diagnosis of the 18 cases of male breast ACC (9–25).

**Table 1:** Clinical features of reported cases of breast adenoid cystic carcinoma in male patients.

No.	Year	Age (years)	Side	Size (cm)	Axillary lymphnode	Treatment	Genetic testing	Follow-up
**1**	1970	37	NM	NM	NM	LE	NM	Recurrence at 5 and 7 years
**2**	1973	78	R	3.5	–	MRM+ALND	NM	Lung metastasis at 9 months
**3**	1974	60	L	NM	-	SM	NM	NM
**4**	1977	21	L	NM	–	SM	NM	Asymptomatic at 24 months
**5**	1991	13	R	3.8	-	SM	NM	Asymptomatic at 30 months
**6**	2006	80	R	4	+,1/10	SM +ALND	NM	Asymptomatic at 60 months
**7**	2006	82	L	NM	+,3/5	MRM+ALND	NM	Recurrence at 24 months
**8**	2012	20	R	2.1	–	SM+SNB	NM	NM
**9**	2012	60	L	1.3	-	RM	NM	Asymptomatic at 24 months
**10**	2013	41	L	1.7	+	NM	NM	Bone and lung metastasis
**11**	2015	19	R	3.0	-	RM+ALND	NM	Asymptomatic at 67 months
**12**	2017	42	L	4.0	–	MRM+ALND	NM	NM
**13**	2019	21	L	5.0	-	SM + SNB	NM	Asymptomatic at 12 months
**14**	2019	44	R	1.2	–	MRM + ALND	NM	Asymptomatic at 26 months
**15**	2020	60	R	2.1	NM	LE	NM	Lung metastasis
**16**	2020	27	L	1.5	–	MRM	MYB+	Asymptomatic at 4 months
**17**	2021	24	L	1.3	-	MRM + ALND	NM	Asymptomatic at 28 months
**18**	2022	37	R	1.5	–	MRM + ALND	MYB-	Asymptomatic at 82 months

NM, not mentioned; R, right; L, left; +, positive axillary lymph node, -, negative axillary lymph node; LE, lumpectomy; MRM, modified radical mastectomy; ALND, axillary lymph node dissection; SM, simple mastectomy; SNB, sentinel node biopsy; RM, radical mastectomy.

### Clinical Characteristics

Similar to the present case, all 17 previously described male patients with breast ACC presented with breast lumps. A portion of patients experienced concomitant pain, and most masses were located beneath the nipple or areola. Among the 18 cases reported to date, the age of onset ranged from 13 to 82 years, with a median age of 39 years. The lesion diameter ranged from 1.2 to 5 cm. Three of the 18 (16.7%) patients tested positive for axillary lymph node metastasis. Simple (6/18) and modified radical mastectomies (7/18) were the most common surgical treatments. Two patients underwent a lumpectomy; a radical mastectomy was performed on two others. 2/15 developed local recurrence and 3 distant metastasis. Both patients who underwent a lumpectomy had a recurrence or distant metastasis after several months and it appears that mastectomy is better surgery than lumpectomy in breast ACC. This may be due to the infiltrative nature of ACC which may result in incomplete excisions.

### Histological Characteristics

Breast and salivary gland ACCs are morphologically similar. Under the microscope, it manifests in three main growth patterns (cribriform, tubular, and solid), which often coexist. Microcystic patterns are also seen occasionally. Double-layered structures comprising glandular epithelial and myoepithelial/basal cells can be found within each tumor pattern, with the glandular epithelial cells of the inner layer exhibiting a cuboidal shape, acidophilic cytoplasm, round nucleus, and occasional nucleoli. In comparison, the myoepithelial/basal cells of the outer layer exhibit a spindle shape, low cytoplasmic volume, intense nuclear staining, and sporadic mitosis. Neural invasion is common (26). In this patient, cribriform structures, myoepithelial/basal cells, and basement membrane materials were observed during microscopic examination. These findings were of diagnostic significance.

### Immunohistochemical and Molecular Genetic Characteristics

In breast ACC, the glandular epithelial cells often express CD117, CK5/6, CK8/18, and CK14 but mostly do not express ER, PR, or HER2. Myoepithelial cells are usually positive for 34βE12, SMA, S-100, and P63. Basement membrane materials can be highlighted by collagen type IV staining. The t (6;9) (q22–23; p23–24) chromosomal translocation frequently occurs in breast ACC, resulting in the fusion of the proto-oncogene *MYB* and the transcription factor gene NFIB (5, 6). Previous studies have shown that *MYB* rearrangements can be detected in approximately 22.6% of breast ACC cases (5, 6). Among the 17 male patients with breast ACC reported in the literature, only two underwent *MYB* gene testing. *MYB-NFIB* gene fusion was detected in one case, but no *MYB* rearrangement was found in another (present) case. Although *MYB* rearrangement was not detected in our case, the presence of the typical histological characteristics, morphology, and immunophenotype of breast ACC served as an adequate basis for diagnosis.

### Differential Diagnosis

#### Cribriform Ductal Carcinoma

Basement membrane materials or basophilic secretions are absent in the structures of cribriform ductal carcinoma. In contrast, basement membrane materials are seen around the cribriform structure of ACC in the male breast with basophilic secretions in the cavity. In addition, only one type of epithelial cell exists within the duct of cribriform ductal carcinoma. In general, myoepithelial/basal cells are arranged successively in a linear pattern around the duct of cribriform ductal carcinoma, which differs from the simultaneous presence of glandular epithelial and myoepithelial/basal cells in the cribriform structures of breast ACC (26).

#### Invasive Cribriform Carcinoma

Invasive cribriform carcinoma (ICC) is often accompanied by tubular carcinoma and is morphologically similar to breast ACC. However, cribriform cell nests of ICC are more irregular than ACC. ICC Cribriform holes contain mucoprotein secretions or necrotic tissue, and there is an absence of acidophilic basement membrane material around the cell nests, whereas basement membrane materials are seen around ACC cell nests. Immunohistochemical markers indicate the absence of myoepithelial/basal cells in ICC, while ACC did not lack myoepithelial/basal cells. In addition, immunohistochemical staining often yields positive results for ER and PR and a negative result for p63 of ICC. However, ER and PR are both negative, and p63 is positive in ACC (26).

#### Microglandular Adenosis

Breast ACC may be misdiagnosed as microglandular adenosis due to the presence of small tubular structures that exhibit invasive growth and contain mucinous secretions (26). However, the glands of microglandular adenosis are covered with a single layer of glandular epithelium, and the lumens contain acidophilic secretions. In atypical cases or cases with carcinogenesis, glandular structures are fused and may exhibit solid growth, but the high nuclear grade and significant mitosis are not found in breast ACC. In addition, immunohistochemical staining of microglandular adenosis indicates the absence of myoepithelial/basal cells, and immunohistochemical p63 negativity can be differentiated from ACC (26).

#### Acinic Cell Carcinoma

Acinic cell carcinoma is an extremely rare disease in which tumor cells form randomly distributed small round glands or solid cell nests with boundaries. These features of acinic cell carcinoma are similar to ACC. Therefore, it is necessary to distinguish it from ACC. However, cells in acinic cell carcinoma contain an abundance of coarse red-stained granules in the cytoplasm, have distinct cell boundaries, irregularly shaped nuclei, and generally lack myoepithelial/basal differentiation, which helps distinguish it from breast ACC (26).

### Treatment and Prognosis

The clinicopathological characteristics of breast cancer differ between males and females. Biological factors, such as anatomical differences and hormone regulation, may result in different responses to treatment (27). Currently, there are no clinical guidelines for treating male breast ACC. Given that wide local excision has been associated with a high postoperative recurrence rate, modified radical mastectomy is generally believed to be required for treatment. The usefulness of radiotherapy and chemotherapy remains unclear, while endocrine therapy is unnecessary in most cases due to ER and PR negativity (3). Compared with other invasive cancers, ACC has a relatively favorable prognosis, with only a few cases of recurrence and metastasis observed during long-term follow-up. Positive margin, Nottingham grade, and neovascularization are associated with the recurrence and distant metastasis of tumors (28). However, the rarity of male breast ACC has resulted in a lack of treatment and prognostic data. Further case reports of male breast ACC are needed for in-depth analyses to understand this disease better.

In conclusion, we reported a case of a male patient with a definitive diagnosis of breast ACC who was treated successfully with modified radical mastectomy. Our patient demonstrated the longest follow-up without tumor recurrence or metastasis among all cases of male breast ACC reported to date. Compared with the commonly used breast-conserving surgery, modified radical mastectomy has a larger surgical scope because it involves axillary lymph node dissection, which may lead to better prognoses. However, this remains under debate (8). Furthermore, the technique has disadvantages due to the large scope of the operation, which may be more distressing for the patient. Therefore, more research is needed to assess and improve therapeutic interventions in breast ACC. Finally, we reviewed the 17 cases previously reported in the literature, discussing the criteria for the differential diagnosis of this rare condition. Furthermore, we found that mastectomies have a better prognosis than lumpectomy.

## Patient Perspective

Despite the postoperative wound infection, our patient was satisfied with this treatment and believes that maintaining an optimistic attitude is as important as selecting an appropriate surgical technique.

## Data Availability Statement

The original contributions presented in the study are included in the article/supplementary material. Further inquiries can be directed to the corresponding author.

## Ethics Statement

The studies involving human participants were reviewed and approved by Ethics Committee of Zigong First People’s Hospital. The patients/participants provided their written informed consent to participate in this study. Written informed consent was obtained from the individual(s) for the publication of any potentially identifiable images or data included in this article.

## Author Contributions

DW and HZ collected the clinicopathological data, searched the literature, and wrote the manuscript. YZ prepared histopathological examination and illustrations. DW and HZ have contributed equally to this work and share first authorship. All authors contributed to the article and approved the submitted version.

## Conflict of Interest

The authors declare that the research was conducted in the absence of any commercial or financial relationships that could be construed as a potential conflict of interest.

## Publisher’s Note

All claims expressed in this article are solely those of the authors and do not necessarily represent those of their affiliated organizations, or those of the publisher, the editors and the reviewers. Any product that may be evaluated in this article, or claim that may be made by its manufacturer, is not guaranteed or endorsed by the publisher.

## References

[1] Miyai K Schwartz MR Divatia MK Anton RC Park YW Ayala AG . Adenoid Cystic Carcinoma of Breast: Recent Advances. World J Clin Cases (2014) 2:732–41. doi: 10.12998/wjcc.v2.i12.732 PMC4266822 25516849

[2] Zhang W Fang Y Zhang Z Wang J . Management of Adenoid Cystic Carcinoma of the Breast: A Single-Institution Study. Front Oncol (2021) 11:621012. doi: 10.3389/fonc.2021.621012 33791208 PMC8005703

[3] Ghabach B Anderson WF Curtis RE Huycke MM Lavigne JA Dores GM . Adenoid Cystic Carcinoma of the Breast in the United States (1977 to 2006): A Population-Based Cohort Study. Breast Cancer Res (2010) 12:R54. doi: 10.1186/bcr2613 20653964 PMC2949643

[4] Defaud-Hénon F Tunon-de-Lara C Fournier M Marty M Velasco V de Mascarel I . Adenoid Cystic Carcinoma of the Breast: Clinical, Histological and Immunohistochemical Characterization. Ann Pathol (2010) 30:7–16. doi: 10.1016/j.annpat.2010.01.003 20223349

[5] D’Alfonso TM Mosquera JM MacDonald TY Padilla J Liu YF Rubin MA . MYB-NFIB Gene Fusion in Adenoid Cystic Carcinoma of the Breast With Special Focus Paid to the Solid Variant With Basaloid Features. Hum Pathol (2014) 45:2270–80. doi: 10.1016/j.humpath.2014.07.013 25217885

[6] Persson M Andrén Y Mark J Horlings HM Persson F Stenman G . Recurrent Fusion of MYB and NFIB Transcription Factor Genes in Carcinomas of the Breast and Head and Neck. Proc Natl Acad Sci USA (2009) 106:18740–4. doi: 10.1073/pnas.0909114106 PMC2773970 19841262

[7] Arpino G Clark GM Mohsin S Bardou VJ Elledge RM . Adenoid Cystic Carcinoma of the Breast: Molecular Markers, Treatment, and Clinical Outcome. Cancer (2002) 94:2119–27. doi: 10.1002/cncr.10455 12001107

[8] Welsh JL Keeney MG Hoskin TL Glazebrook KN Boughey JC Shah SS . Is Axillary Surgery Beneficial for Patients With Adenoid Cystic Carcinoma of the Breast? J Surg Oncol (2017) 116:690–5. doi: 10.1002/jso.24702 28608456

[9] Woyke S Domagala W Olszewski W . Fine Structure of Mammary Adenoid Cystic Carcinoma. Pol Med J (1970) 9:1140–8. 4324184

[10] Verani RR van der Bel-Kahn J . Mammary Adenoid Cystic Carcinoma With Unusual Features. Am J Clin Pathol (1973) 59:653–8. doi: 10.1093/ajcp/59.5.653 4349849

[11] Ferlito A Di Bonito L . Adenoid Cystic Carcinoma of the Male Breast: Report of a Case. Am Surg (1974) 40:72–6. 4357545

[12] Hjorth S Magnusson PH Blomquist P . Adenoid Cystic Carcinoma of the Breast. Report of a Case in a Male and Review of the Literature. Acta Chir Scand (1977) 143:155–8. doi: 10.1038/nn1304 200041

[13] Miliauskas JR Leong AS . Adenoid Cystic Carcinoma in a Juvenile Male Breast. Pathology (1991) 23:298–301. doi: 10.3109/00313029109063592 1664513

[14] Maciag H Ziolkowski P Wrzecion S Kolodziej P . Adenoid Cystic Carcinoma of the Breast in an 80-Year-Old Male - a Case Report. Wspolczesna Onkol (2006) 10:340. Available at: https://www.termedia.pl/Adenoid-cystic-carcinoma-of-the-breast-in-an-80-year-old-male-8211-a-case-report,3,6594,1,1.html.

[15] Kshirsagar AY Wader JV Langade YB Jadhav KP Zaware SU Shekhar N . Adenoid Cystic Carcinoma of the Male Breast. Int Surg (2006) 91:234–6. 16967686

[16] Liu J Jia W Zeng Y Deng H Rao N Su F . Adolescent Male Adenoid Cystic Breast Carcinoma. Am Surg (2012) 78:288–9. doi: 10.1177/000313481207800519 22691333

[17] Sahan EK Karinoglu U Igdem AA Erdogan N . Adenoid Cystic Carcinoma in Male Breast: A Case Report. Virchows Arch (2012) 461(Suppl 1):S244.

[18] Yoo SJ Lee DS Oh HS Kim HJ Kim MH Ahn YC . Male Breast Adenoid Cystic Carcinoma. Case Rep Oncol (2013) 6:514–9. doi: 10.1159/000356062 PMC3884210 24403896

[19] Tang P Yang S Zhong X Yao J Zhang Y Dong H . Breast Adenoid Cystic Carcinoma in a 19-Year-Old Man: A Case Report and Review of the Literature. World J Surg Oncol (2015) 13:19. doi: 10.1186/s12957-015-0442-8 25885366 PMC4329652

[20] Yahya ZH Aminmozaffari S Vand RM . Male Adenoid Cystic Carcinoma of Breast. Int J Cancer Manag (2017) 10:e7827. doi: 10.5812/ijcm.7827

[21] Mackenzie J Douglas C . Gynecomastia or Rare Malignancy? A Young Man With an Unremarkable History Proves to Have Adenoid Cystic Carcinoma of the Breast. Breast J (2020) 26:502–4. doi: 10.1111/tbj.13534 31493308

[22] Pang W Wang Z Jin X Zhang Q . Adenoid Cystic Carcinoma of the Breast in a Male: A Case Report. Medicine (2019) 98:e16760. doi: 10.1097/MD.0000000000016760 31393393 PMC6708915

[23] Hogan KO Fan F . Diagnosis of Metastatic Adenoid Cystic Carcinoma of the Breast on Pleural Fluid Cytology in a 60-Year-Old Male. Diagn Cytopathol (2021) 49:E172–4. doi: 10.1002/dc.24636 33035408

[24] Leilei L Rusong Z Xuan W Nan W Rui L Kai C . Adenoid Cystic Carcinoma of Male Breast: A Clinicopathological Study. Chin J Diagn Pathol (2020) 27:775–8. doi: 10.3969/j.issn.1007-8096.2020.11.002.

[25] Li JX Zhang XM Xiao YX Tang ZM Huang T Ming J . Male Adenoid Cystic Carcinoma of the Breast. J Med cases (2021) 12:503–10. doi: 10.14740/jmc3790 PMC8683108 34970375

[26] Stuart JS Laura CC . Biopsy Interpretation of the Breast. 3rd ed. USA: Wolters Kluwer Health Press (2018).

[27] Lin AP Huang TW Tam KW . Treatment of Male Breast Cancer: Meta-Analysis of Real-World Evidence. Br J Surg (2021) 108:1034–42. doi: 10.1093/bjs/znab279 34476472

[28] Slodkowska E Xu B Kos Z Bane A Barnard M Zubovits J . Predictors of Outcome in Mammary Adenoid Cystic Carcinoma: A Multi-Institutional Study. Am J Surg Pathol (2020) 44:214–23. doi: 10.1097/PAS.0000000000001378 31567278

